# Association of Nadir Prostate-specific Antigen >0.5 ng/mL after Dose-escalated External Beam Radiation with Prostate Cancer-specific Endpoints

**DOI:** 10.7759/cureus.2790

**Published:** 2018-06-12

**Authors:** Niki Sheth, Irini Youssef, Virginia Osborn, Anna Lee, Joseph Safdieh, David Schreiber

**Affiliations:** 1 Radiation Oncology, SUNY Downstate Medical Center, New York, USA; 2 Radiation Oncology, SUNY Downstate Medical Center & Veterans Affairs, New York Harbor Healthcare System; 3 Radiation Oncology, Kings County Hospital Center; 4 Radiation Oncology, New York Harbor Healthcare System

**Keywords:** prostate adenocarcinoma, prostate cancer, psa, psa nadir, prostate cancer-specific endpoints, high-risk prostate cancer, intermediate-risk prostate cancer

## Abstract

Objective

Prior studies have suggested that prostate-specific antigen (PSA) nadir of 0.5 ng/mL is an important surrogate endpoint for prostate cancer-specific and all-cause mortality. This study analyzed our well-followed patient cohort to assess whether this endpoint was associated with differences in prostate cancer-specific endpoints in patients receiving dose-escalated radiation.

Methods

Patients with intermediate- or high-risk prostate cancer (≥T2b, or prostate-specific antigen >10 ng/mL, or Gleason score ≥7) who were treated with external beam radiation to a minimum dose of 7560 cGy +/- androgen deprivation between 2003 and 2011 were identified. Biochemical control, distant metastasis-free survival (DMFS), prostate cancer-specific survival (PCSS), and overall survival (OS) were compared between those who achieved a nadir PSA ≤0.5 ng/mL with those who did not via Kaplan-Meier analysis. Univariable and multivariable Cox regression was performed on all endpoints to assess their impact on OS.

Results

There were 367 patients identified with a median follow-up of 99.5 months. Two hundred five patients (55.9%) received androgen deprivation for a median of 24 months (range 1-81 months). Most patients (n = 308, 83.9%) achieved a nadir PSA ≤0.5 ng/mL, which was associated with improvement across all endpoints at 10 years. This included biochemical control (68.0% versus 24.0%, p < 0.001), DMFS (89.6% versus 80.8%, p = 0.019), PCSS (91.1% versus 85.7%, p = 0.01), and OS (55.7% versus 45.8%, p = 0.048). On multivariable analysis, nadir PSA >0.5 ng/mL remained strongly associated with worse outcomes across all endpoints.

Conclusions

Achievement of nadir PSA ≤0.5 ng/mL after completion of dose-escalated radiation therapy was associated with improvement of all prostate cancer endpoints.

## Introduction

Prostate cancer is the most common genitourinary cancer with a lifetime risk estimated to be approximately one in six for men in the United States [1]. In 2017 alone, there were an estimated 161,000 cases and 26,700 deaths, representing 9.6% of all new cancer diagnoses [2].

There are several definitive treatment options available for prostate cancer including external beam radiation therapy (EBRT), brachytherapy, or surgery. Some patients may also receive androgen deprivation therapy (ADT). Regardless of treatment modality employed, patients are recommended to be closely monitored for response to treatment and possible progression or recurrence.

After completion of radiation therapy (RT), biochemical failure is generally defined as a 2.0 ng/mL or higher rise in prostate-specific antigen (PSA) above the PSA nadir [3]. However, this definition does not always translate to other important clinical endpoints, such as metastases and prostate cancer-specific death [4]. Despite a biochemical recurrence, a patient may die of any one of multiple competing risks and not necessarily due to prostate cancer. As such, several studies have sought to identify other prognostic PSA values that can be used as surrogates for these clinical endpoints.

Some studies have suggested that a PSA nadir of >0.5 ng/mL after conventional dose RT with or without ADT is an important surrogate endpoint for prostate cancer-specific and all-cause mortality [5-7]. However, only one of these studies included patients treated with dose-escalated radiation therapy and the median follow-up was only three years. In the current study, we analyzed our well-followed patient cohort with intermediate- or high-risk prostate cancer who was treated with ≥7560 cGy to assess whether PSA nadir >0.5 ng/mL predicted for differences in all prostate cancer endpoints.

## Materials and methods

After institutional review board approval, we retrospectively reviewed the charts of 367 consecutive men who were diagnosed with intermediate- or high-risk prostate cancer and treated with definitive external beam radiation (minimum dose 7560 cGy) between 2003 and 2011. The radiation techniques have been previously described [8]. Briefly, all patients were treated supine and immobilized in a thermoplastic cast. Treatments were delivered via three-dimensional conformal radiation (2003-2006), intensity modulated radiation therapy without image guidance (2006-2009), or intensity modulated radiation therapy with image guidance via a daily megavoltage cone beam computed tomography scans matched either to the bony anatomy or to gold fiducial markers (2010-2011). The radiation fields varied based on the National Cancer Care Network (NCCN) risk group but typically included treatment of the pelvic lymph nodes for high-risk disease and prostate plus seminal vesicles only for intermediate-risk disease.

Patients who received androgen deprivation were treated with a luteinizing hormone agonist. The length of the androgen deprivation was also based on physician discretion but was generally six months for intermediate-risk and two to three years for high-risk disease. Upon completion of treatment, patients were generally followed every three to six months for five years, followed by yearly PSA checks. Biochemical failure was defined using the definition of PSA nadir + 2.0 ng/mL. If the cause of death was unknown and the patient was known to have metastatic disease at the time of recurrence, it was recorded in our database as a prostate cancer-related death. If the patient had no evidence of disease at the time of death but the precise cause of death was unknown, it was recorded as unknown.

Patient characteristics were compared using Chi-Square, Fisher’s Exact test, and Mann-Whitney U where appropriate. Biochemical failure-free survival (bFFS), distant metastatic progression-free survival (DMPFS), prostate cancer-specific survival (PCSS) and overall survival (OS) were determined from the date of completion of radiation treatments and were stratified based on whether they achieved a PSA nadir of ≤0.5 ng/mL or >0.5 ng/mL. Their outcomes were analyzed using the Kaplan‑Meier method and compared using the log‑rank test. Univariable and multivariable Cox regression modeling was performed to determine their impact on the measured outcomes. These variables included age (continuous), race (White, Black, Hispanic), PSA grouping (≤10 ng/mL, 10-20 ng/mL, >20 ng/mL), Gleason score (6, 7, 8-10), androgen deprivation (no, yes), and nadir PSA value (≤0.5 ng/mL, >0.5 ng/mL). Statistical analysis was performed using the statistical package for the social sciences (SPSS) version 23.0 (IBM Inc, Armonk, New York) and statistical significance was achieved with a p-value < 0.05.

## Results

There were 367 patients included in this study, with 236 (64.4%) alive at last follow-up. The median follow-up for living patients was 99.5 months (interquartile range 72-121 months). The median age at the time of radiation treatment was 71 years (interquartile range 62-76 years). Most men (n = 228, 62.1%) were Black, followed by 109 (29.7%) White and 30 (8.2%) Hispanic men. The most common radiation dose was 7560 cGy (n = 274, 74.7%). Intermediate-risk disease comprised 212 patients (57.8%) and 155 men (42.2%) had high-risk disease. Most men with intermediate-risk disease did not receive androgen deprivation (n = 146, 68.9%) whereas most patients with high-risk disease did receive androgen deprivation (n = 139, 89.7%). The median duration of androgen deprivation was 24 months (interquartile range 6-27 months). Further details regarding patient characteristics and a comparison between those who did or did not achieve a PSA nadir of <0.5 ng/mL are available in Table 1.

**Table 1:** Patient characteristics and comparison between groups. PSA: Prostate-specific antigen; RT: Radiation therapy; 3D-CRT: Three-dimensional conformal radiation therapy; IMRT: Intensity-modulated radiation therapy.

	PSA nadir <0.5 ng/mL (n = 308)	PSA nadir >0.5 ng/mL (n = 59)	p-value
Age (median)	71 years	68 years	0.08
Race			0.64
White	91 (83.5%)	18 (16.5%)	
Black	190 (83.3%)	38 (16.7%)	
Hispanic	27 (90.0%)	3 (10.0%)	
Hormones			<0.001
No	109 (67.3%)	53 (32.7%)	
Yes	199 (97.1%)	6 (2.9%)	
Initial PSA			0.09
≤10 ng/mL	162 (81.4%)	37 (18.6%)	
10-20 ng/mL	91 (83.5%)	18 (6.5%)	
>20 ng/mL	55 (93.2%)	4 (6.8%)	
Gleason			0.10
6	34 (81.0%)	8 (190%)	
7	173 (81.2%)	40 (18.8%)	
8-10	101 (90.2%)	11 (9.8%)	
RT technique			0.25
3D-CRT	176 (81.9%)	39 (18.1%)	
IMRT	132 (86.8%)	20 (13.2%)	
RT dose			1.0
75.6 Gy	230 (83.9%)	44 (16.1%)	
77.4-81 Gy	78 (83.9%)	15 (16.1%)	

Biochemical control

**Figure 1:**
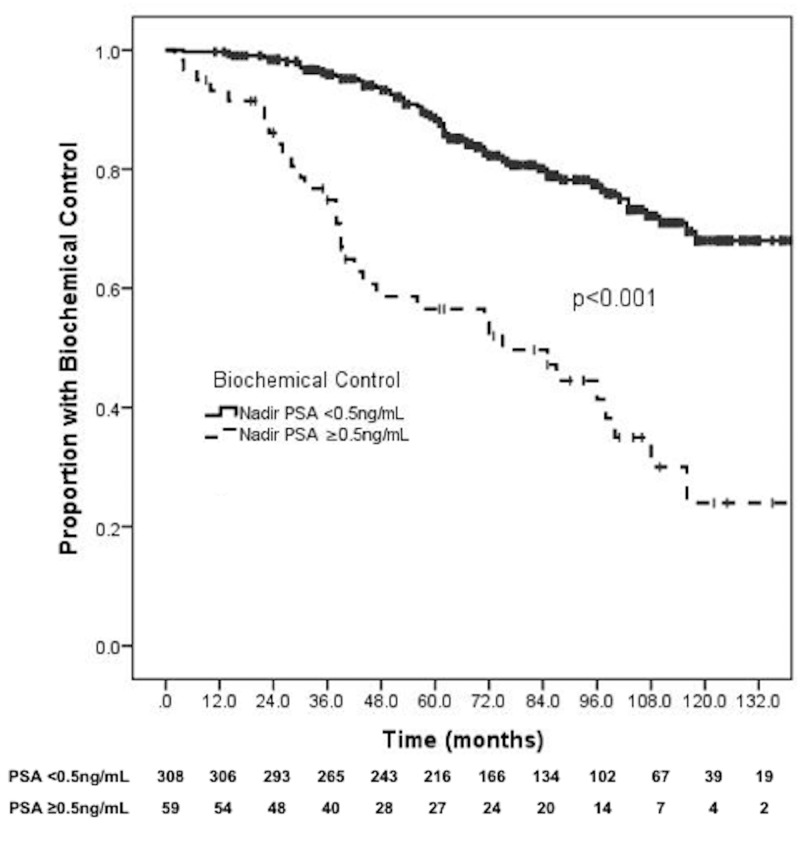
PSA nadir and biochemical control. PSA: Prostate-specific antigen

The 10-year biochemical control was 68.0% for nadir PSA ≤0.5 ng/mL versus 24.0% for nadir PSA >0.5 ng/mL (p < 0.001) (Figure 1). On multivariable analysis, PSA nadir >0.5 ng/mL was the strongest predictor for biochemical failure (Hazard ratio (HR): 5.82, 95% Confidence interval (CI): 3.30-10.28, p < 0.001). Other variables associated with biochemical failure included Gleason score 8-10 (HR: 3.42, 95% CI: 1.68-10.04, p = 0.002) and PSA >20 ng/mL (HR: 3.42, 95% CI: 1.85-6.32, p < 0.001). Further details are available in Table 2.

**Table 2:** Multivariable analysis for biochemical progression-free survival and distant metastatic progression-free survival. HR: Hazard ratio; CI: Confidence interval; PSA: Prostate-specific antigen.

	Biochemical failure		Distant metastatic disease	
	HR (95% CI)	p-value	HR (95% CI)	p-value
Age (continuous)	1.02 (0.99-1.05)	0.08	1.07 (1.02-1.12)	0.008
Race				
White	1		1	
Black	1.07 (0.68-1.68)	0.78	1.09 (0.48-2.48)	0.84
Hispanic	0.43 (0.15-1.26)	0.13	0.58 (0.12-2.78)	0.50
Gleason				
6	1			
7	2.18 (0.90-5.27)	0.09	0.89 (0.18-4.34)	0.88
8-10	4.10 (1.68-10.04)	0.002	4.56 (0.996-20.9)	0.051
PSA				
≤10 ng/mL	1		1	
10-20 ng/mL	1.14 (0.67-1.94)	0.63	0.57 (0.21-1.52)	0.26
>20 ng/mL	3.42 (1.85-6.32)	<0.001	1.27 (0.48-3.37)	0.63
Hormones				
No	1		1	
Yes	0.85 (0.46-1.59)	0.62	3.15 (0.94-10.61)	0.06
Nadir PSA ≤0.5 ng/mL				
Yes	1		1	
No	5.82 (3.30-10.28)	<0.001	10.06 (3.38-30.0)	<0.001

Distant control

**Figure 2:**
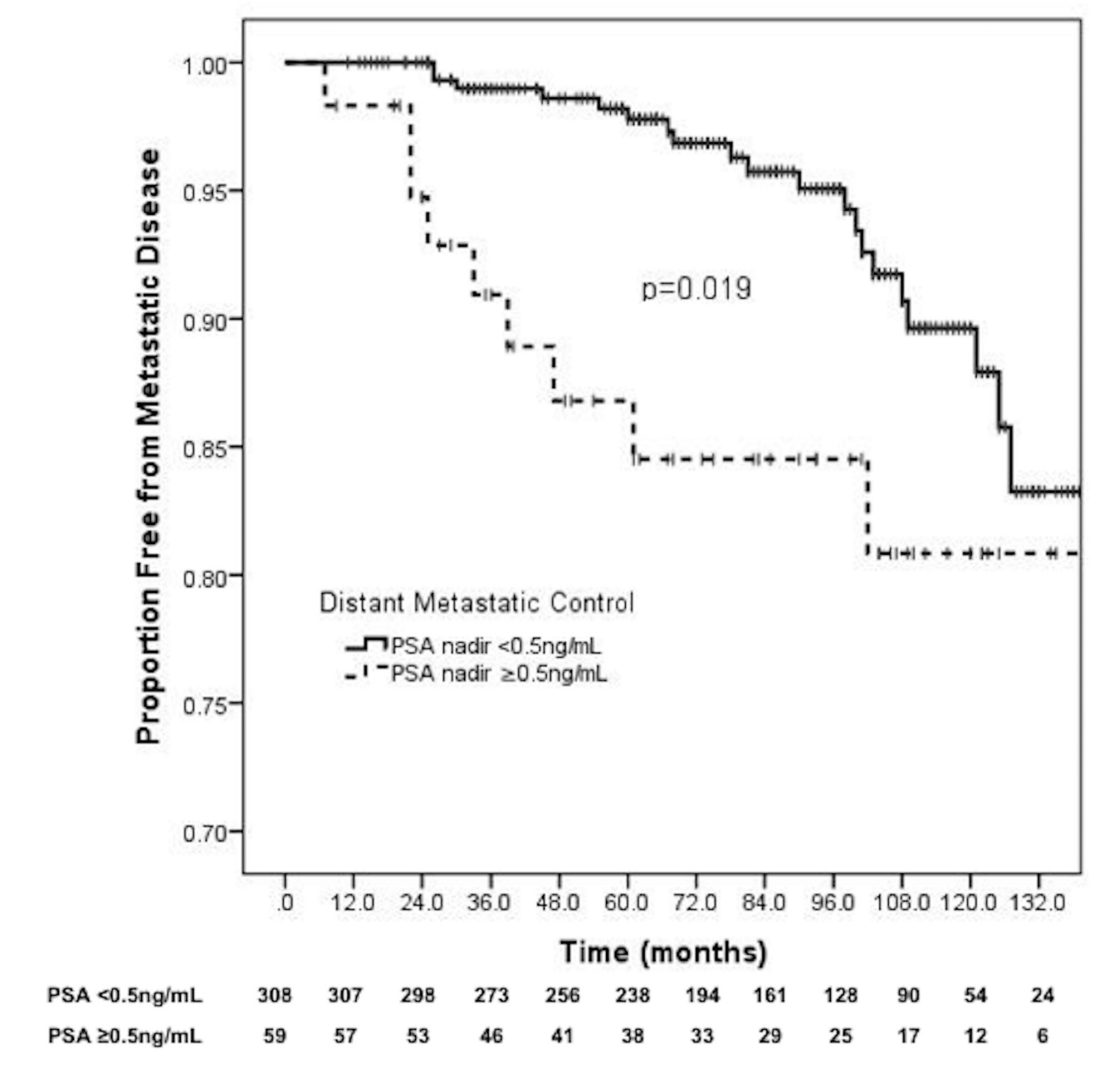
PSA nadir and distant metastatic control. PSA: Prostate-specific antigen

The 10-year distant metastasis-free survival was 89.6% for PSA ≤0.5 ng/mL versus 80.8% for PSA >0.5 ng/mL (p = 0.019) (Figure 2). On multivariable analysis, nadir PSA was a very strong predictor for distant metastases (HR: 10.06, 95% CI: 3.38-30.0, p < 0.001). However, Gleason score 8-10 and PSA value >20 ng/mL were not associated with an increase in distant metastases (Table 2).

Prostate cancer-specific survival and overall survival

**Figure 3:**
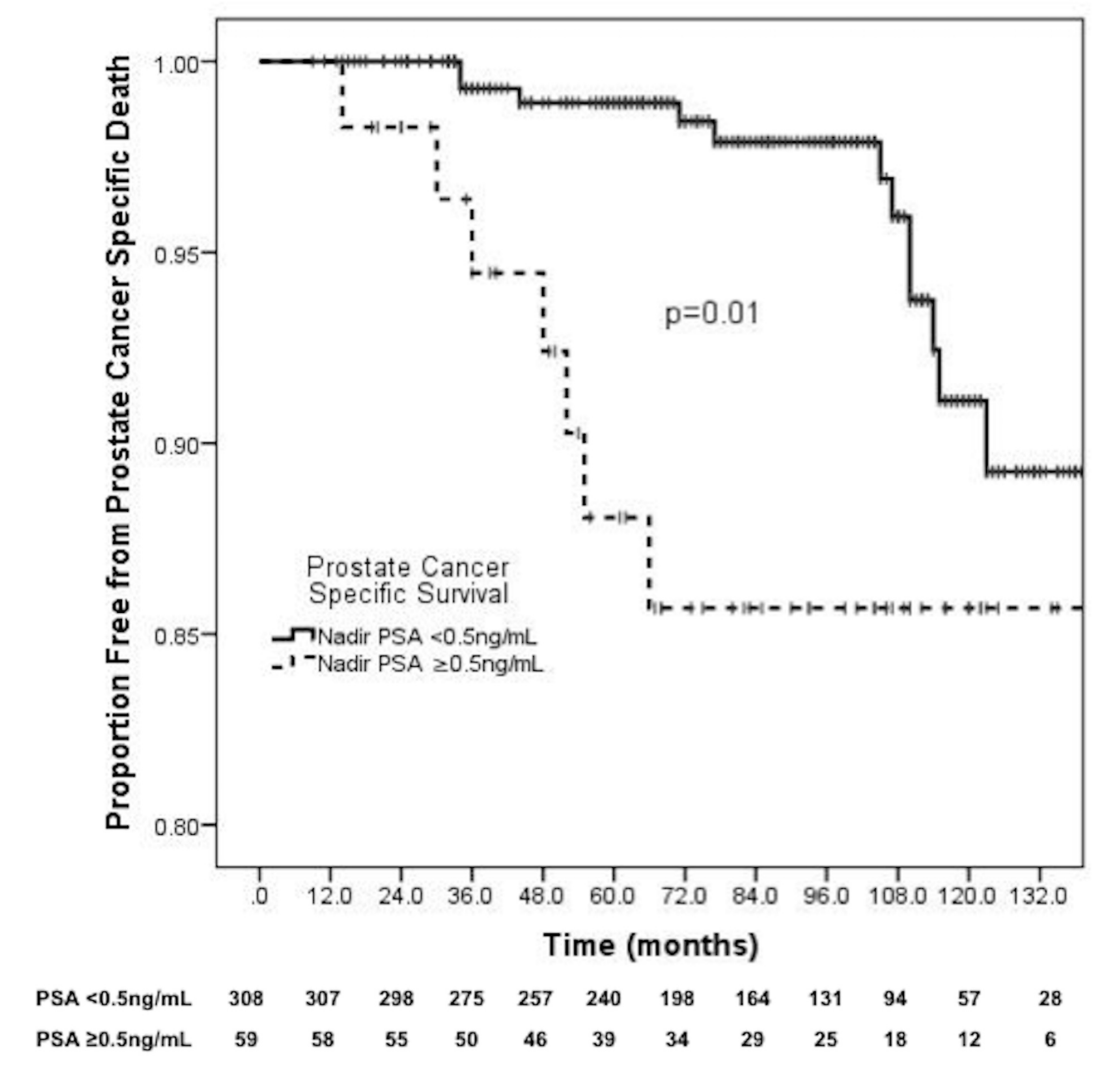
PSA control and prostate cancer-specific survival. PSA: Prostate-specific antigen

**Figure 4:**
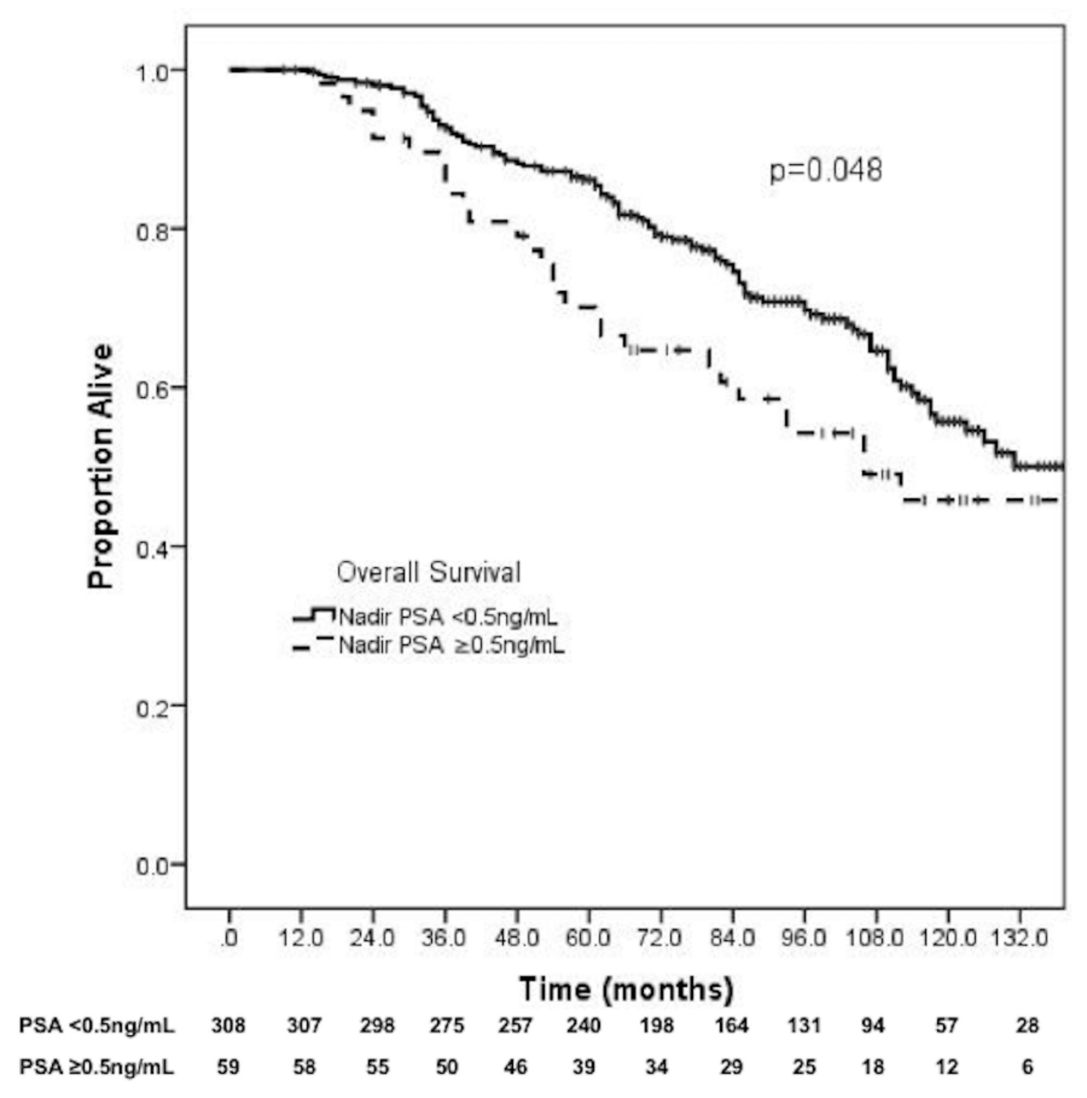
PSA nadir and overall survival. PSA: Prostate-specific antigen

The 10-year PCSS was 91.1% for nadir ≤0.5 ng/mL versus 85.7% for PSA >0.5 ng/mL (p = 0.01) (Figure 3). The 10-year OS was 55.7% for nadir PSA ≤0.5 ng/mL versus 45.8% for nadir PSA >0.5 ng/mL (p = 0.048) (Figure 4). On multivariable analysis, nadir PSA >0.5 ng/mL was strongly associated with an increased likelihood of prostate cancer-specific mortality (HR: 25.70, 95% CI: 5.97-110.52, p < 0.001) and all-cause mortality (HR: 2.94, 95% CI: 1.76-4.91, p < 0.001). Use of androgen deprivation was also associated with a higher likelihood of prostate cancer-specific death (HR: 12.08, 95% CI: 1.87-78.03, p = 0.009). Further details are shown in Table 3.

**Table 3:** Multivariable analysis for prostate cancer-specific mortality and all-cause mortality. HR: Hazard ratio; CI: Confidence interval; PSA: Prostate-specific antigen.

	Prostate cancer-specific mortality		All-cause mortality	
	HR (95% CI)	p-value	HR (95% CI)	p-value
Age (continuous)	1.11 (1.04-1.19)	0.003	1.07 (1.04-1.09)	<0.001
Race				
White	1		1	
Black	1.25 (0.44-3.57)	0.68	1.12 (0.76-1.63)	0.57
Hispanic	0.63 (0.07-5.67)	0.68	0.55 (0.24-1.24)	0.15
Gleason				
6	1			
7	1.84 (0.21-15.81)	0.58	1.14 (0.62-2.08)	0.68
8-10	4.05 (0.47-35.0)	0.20	1.66 (0.88-3.11)	0.12
PSA				
≤10 ng/mL	1		1	
10-20 ng/mL	0.82 (0.25-2.72)	0.75	1.27 (0.84-1.93)	0.26
>20 ng/mL	1.03 (0.27-3.95)	0.96	1.38 (0.82-2.31)	0.22
Hormones				
No	1		1	
Yes	12.08 (1.87-78.03)	0.009	1.64 (1.0-2.68)	0.05
Nadir PSA ≤0.5 ng/mL				
Yes	1		1	
No	25.70 (5.97-110.52)	<0.001	2.94 (1.76-4.91)	<0.001

## Discussion

In our study, we retrospectively analyzed the outcomes of 367 patients treated with dose-escalated radiation therapy for intermediate- and high-risk prostate cancer between the years 2003 and 2011. We found that PSA nadir >0.5 ng/mL is significantly associated with not only biochemical failure (HR: 5.82, p < 0.001) but also distant metastases at 10 years (HR: 10.06, p < 0.001), prostate cancer-specific mortality (HR: 25.70, p < 0.001) and all-cause mortality (HR: 2.94, p < 0.001) at 10 years.

Several studies have previously looked at the relationship between PSA nadir and biochemical disease-free survival with varying PSA nadir cutoffs. Lee et al. studied 364 men with T1-T3 prostate adenocarcinoma who received definitive external beam radiation therapy >68 Gy and followed them for a minimum of 24 months with serial PSA measurements [9-10]. The study found that PSA nadir <1.0 ng/mL was correlated with favorable biochemical disease-free survival which was defined as PSA of 1.5 ng/mL or more with two consecutive PSA elevations.

There have also been studies specifically evaluating PSA nadir of >0.5 ng/mL and biochemical control. Zelefsky et al. evaluated 213 patients with localized prostate cancer in order to identify prognostic variables that could predict for improved biochemical and local control in patients treated with neoadjuvant ADT and three-dimensional conformal radiation therapy (3D-CRT) with median dose of 75.6 Gy [6]. Similar to our study, they found that PSA nadir ≤0.5 ng/mL with PSA relapse-free survival rate of 74% in patients with PSA nadir ≤0.5 ng/mL compared to 40% in those with a higher PSA nadir. A similar retrospective analysis by Zietman et al. analyzed 314 men treated with EBRT to >68.4 Gy for T1-2 disease and discovered an even higher rate of freedom from biochemical progression, 90%, was correlated with PSA nadir ≤0.5 ng/mL [11]. Though these helped establish PSA nadir of ≤0.5 ng/mL as an important predictor of biochemical control, its correlation with other endpoints needed further elucidation.

Ray et al. went further and looked at not only biochemical control but also distant metastases. The study separated 4,839 patients treated with EBRT to doses >60 Gy, into groups using nadir PSA outpoints of 0.5–2.0 ng/mL [12]. Similar to the previously mentioned studies, authors of this study reported an 8-year PSA-disease free survival rate of 75%, 52%, 41%, and 18% (p < 0.0001) for patients with nadir PSA levels of 0–0.49, 0.5–0.99, 1.0–1.99, and 2.0 ng/mL, respectively. However, they also found that lower nadir PSA levels were associated with improvement in metastasis-free survival. For patients with a PSA nadir of 0–0.49, 0.5–0.99, 1.0–1.99, and 2.0 ng/mL, the 8-year distant metastasis-free survival (DMFS) rates were 97%, 96%, 91%, and 73%, respectively (p < 0.0001). Of note, this study used a range of doses including ≥60 Gy and did not include men receiving ADT precluding its utility to extrapolate to high-risk men who receive bimodal treatment. However, we did note similar findings in our dose-escalated patients with a 10-year DMFS of 89.6% for PSA nadir ≤0.5 ng/mL, versus 80.8% for PSA nadir >0.5 ng/mL.

Hanlon et al. at Fox Chase Cancer Center were the first to extend the association between PSA nadir and distant failure to PCSS. The investigators looked at 615 men treated to median dose of 73 Gy with median follow-up of 64 months and found PSA nadir ≥1.0 ng/mL to be highly predictive of distant failure as well as death from prostate cancer [13]. More recently, D’Amico et al. demonstrated a relationship specifically between PSA nadir >0.5 ng/mL and PCSS [14]. They conducted a meta-analysis of two randomized control trials (Dana Farber Cancer Institute (DFCI) trial and the Trans-Tasman Radiation Oncology Group (TROG) trial) on 363 men that used radiotherapy doses ranging from 66 to 70 Gy and ADT versus no ADT [15-16]. The authors reported a PCSS HR of 5.35 (p = 0.0016) in the DFCI trial and 6.17 (p < 0.0001) in the TROG trial in men with a PSA nadir >0.5 ng/mL versus ≤0.5 ng/mL (6). The present report adds to these aforementioned studies in extending these findings to patients receiving dose-escalated radiation therapy as well.

Finally, Royce et al. demonstrated an association between PSA nadir >0.5 ng/mL and all-cause mortality [5]. The investigators analyzed their randomized trial of 206 men with unfavorable-risk prostate cancer (PSA >10 but <40 ng/mL or Gleason score ≥7; or endorectal magnetic resonance imaging evidence of extracapsular extension and/or seminal vesicle invasion) who received 70.2 Gy 3D-CRT alone versus 70.2 Gy and six months of ADT. The authors found PSA nadir >0.5 ng/mL was significantly associated with all-cause mortality (HR: 1.72; p = 0.01). The authors also found a PSA nadir ≤0.5 ng/mL was achieved more often in those who received RT with ADT over RT alone (94% versus 33%, p < 0.001). This was reinforced by our study in which PSA nadir ≤0.5 ng/mL was achieved in 97% of patients who received ADT versus 67% of those who received RT alone.

By defining PSA nadir >0.5 ng/mL as a strong prognostic factor, the findings from this study can help future investigations to determine whether these patients should receive aggressive interventions in the adjuvant setting rather than waiting for the appearance of distant metastases. For example, Sandler et al. reported the results of RTOG 0521 which showed the potential benefit of adding adjuvant docetaxel for non-metastatic, hormone-sensitive prostate cancer. According to the abstract, the group receiving docetaxel had a lower four-year overall survival (93% versus 89%, p = 0.04) and higher disease-free survival (73% versus 66%, p = 0.05) [17]. Another similar study added abiraterone in the treatment of locally advanced or metastatic prostate cancer and showed lower number of deaths (HR: 0.63, p < 0.001) and treatment-failure events (HR: 0.29, p < 0.001) compared to the group without abiraterone [18].

There are several limitations to this study, mostly inherent in its retrospective nature. Patients who received androgen deprivation likely had an increased burden of disease and more aggressive disease. However, 97% of patients that received ADT reached PSA nadir ≤0.5 ng/mL as opposed to only 67% of those who received RT alone. Another limitation was the variation in the androgen deprivation duration as well whether pelvic or prostate fields were used. Though the present study has a long follow-up with median of 99.5 months, it is possible that longer follow-ups with more events are still needed to get a more accurate assessment of the impact of this PSA nadir value. Finally, although this is a fairly large study of 367 patients, a larger study with more events may be needed to confirm these results.

## Conclusions

We found that a nadir PSA >0.5 ng/mL after completion of ≥7560 cGy +/- androgen deprivation was associated with a higher risk of biochemical failure, distant metastatic failure, prostate cancer-specific mortality, and all-cause mortality. These patients should be considered the targets for aggressive adjuvant therapy in future prospective studies.
